# Emotional intelligence and emotion information processing: Proof of concept of a test measuring accuracy in discriminating emotions

**DOI:** 10.3389/fpsyg.2023.1085971

**Published:** 2023-02-03

**Authors:** Christelle Gillioz, Maroussia Nicolet-dit-Félix, Oliver Wilhelm, Marina Fiori

**Affiliations:** ^1^Research and Development, Swiss Federal University for Vocational Education and Training, Renens, Switzerland; ^2^Department of Individual Differences and Psychological Assessment, Ulm University, Ulm, Germany

**Keywords:** ability-EI, emotion blends, emotional intelligence, hypersensitivity, emotion information processing, emotion discrimination, emotion recognition

## Abstract

Emotion information processing (EI_P_) has been recently introduced as a new component of emotional intelligence. We present a task aiming at measuring a type of emotion information processing related to fine-grained discrimination of emotional expressions. We modified an existing task presenting morphed faces created from a blend of two prototypical emotional expressions. Participants’ (*N* = 154) ability-EI, in particular emotion recognition, understanding and management, as well as intelligence were evaluated. Results show that all facets of EI independently predicted accuracy in the discrimination task and that emotion recognition was the strongest predictor. When controlling for emotion recognition level, we found that emotion understanding still predicted accuracy for less difficult stimuli. Results support the idea that individuals high in EI have higher emotion processing skills at the emotion perception stage of information processing and suggest that the task employed in the current study might measure more spontaneous processing of emotional expressions. Implications regarding the use of the current task as a new measure of the EI_P_ component are discussed.

## 1. Introduction

Emotional intelligence (EI) corresponds to the skills related to the perception, understanding and management of emotion. Two major conceptualizations of EI are present in the scientific literature. The first one, trait-EI, defines EI as dispositions or personality characteristics that explain how individuals behave in emotional situations (Petrides and Furnham, 2001). The second one, ability-EI, views EI as an ability related to the processing of emotional information (Mayer et al., 2016). Whereas trait-EI is measured with self-report questionnaires, ability-EI is assessed with performance tests designed at evaluating each EI facet (emotion recognition, understanding and management). For instance, the Situational Test of Emotion Understanding (STEU; MacCann and Roberts, 2008) presents descriptions of short emotional scenarios and respondents have to select the appropriate emotion or indicate which event lead to a specific emotion.

Recently, it has been proposed that ability-EI is not a monolithic construct, but that it is likely based on two components: (1) the emotion knowledge component (EI_K_) and (2) the emotion processing component (EI_P_) (Fiori et al., 2022). EI_K_ is related to higher order reasoning or top–down processing about emotions and corresponds to what is habitually measured with performance-based ability-EI tests, i.e., knowledge about emotions. EI_P_ is related to bottom-up processing about emotion and can be assessed with emotion processing tasks, evaluating more spontaneous and fast processing of emotion information. Drawing a parallel with intelligence (Cattell, 1963), EI_K_ is conceptualized as a crystallized component of EI, related to culture-bound knowledge about emotion, and EI_P_ as a fluid component of EI, related to how people feel and experience emotion (Fiori and Vesely-Maillefer, 2018; Fiori et al., 2022). EI_K_ and EI_P_, while being different constructs, are nonetheless related: individuals high on EI_K_ should also be high on EI_P_. In other terms, with high EI, individuals should not only demonstrate more emotional knowledge and perform better at ability-EI tests, but also more efficiently process emotional stimuli in a spontaneous manner.

The inclusion of the EI_P_ component in the conceptualization of EI allows us to offer an explanation as to how EI functions regarding emotional and cognitive processes that are taking place in high vs. low-EI individuals. Previous research has indeed suggested that individuals high in EI are more efficient in tasks with emotionally laden stimuli (Gutiérrez-Cobo et al., 2016, 2017). In addition, the *hypersensitivity hypothesis* (Fiori and Ortony, 2021) states that EI works as a magnifier of emotional experience. In this view, high-EI individuals are hypersensitive to emotion information, which can be observed at different stages of emotion processing. High-EI_P_ individuals are then expected to better perceive and encode emotion, to experience more intense emotional reactions, and to show greater attention to emotional stimuli. This was demonstrated in a recent study (Nicolet-dit-Félix et al., 2023) in which individuals high on the emotion understanding facet of EI showed an attentional bias to emotional faces in a dot-probe task, in which they had to identify a letter appearing at the location of an emotional vs. a neutral face. The difference in response times between the conditions was apparent for individuals scoring above 1 standard deviation from the mean, supporting the ideas that EI_P_ increases with EI_K_ and that hypersensitivity toward emotional information appears at high levels of EI.

Importantly and as said above, the fluid component of EI, EI_P_, is not captured by current ability-EI tests, which tap into general knowledge about emotions. These tests, by their very instructions and design, measure the respondent’s maximum-ability performance, which may not correspond to their actual emotional behavior (Fiori, 2009). For example, it is possible to know how to manage one’s own emotions in different situations, hence obtain a high score in an emotion management ability test, without being capable of doing so in a real situation. In addition, whereas current ability-EI tests measure broad facets, namely perceiving, understanding and managing emotions, EI_P_ is concerned about the underlying processes accounting for such facets, such as attentional processes. Finally, ability-EI tests rely on conscious processing of emotion information, whereas EI_P_ is meant to capture more spontaneous and automatic reactions to emotion information (Fiori, 2009). There is therefore a need to develop measures of EI_P_ in order to consider this component when investigating the role of EI on life outcomes. For instance, it has been shown that high levels of EI, particularly the emotion perception facet, can lead to higher levels of stress during stressful situations (Matthews et al., 2006; Bechtoldt and Schneider, 2016). This kind of finding is difficult to explain based solely on the EI_K_ component. However, if we consider individual differences in how people process emotion information and include EI_P_ in the equation, then these findings could be interpreted as reflecting hypersensitivity to emotions (i.e., individuals high in EI pay more attention or better discriminate emotions in their surroundings which can lead to higher stress).

Previous research examining EI_P_ has focused on the attentional processes related to emotion processing and has employed experimental tasks tapping into such processes. For example, Fiori et al. (2022) used an emotional Stroop task and a GonoGo task to operationalize EI_P._ They showed that scores in these tasks predicted additional variability (i.e., above the one predicted by ability-EI tests) in emotionally intelligent behavior. EI_P_ is nonetheless not only related to attentional processes, but also concerns other types of processes related to the three broad facets of EI. In this article, we aim at offering a way to measure EI_P_ mainly related to the facet of emotion perception and to investigate how hypersensitivity at the level of fine-grained discrimination of emotions is related to EI_K_.

Emotion perception is considered the basis of EI (Mayer et al., 2008). For example, the cascading model of EI considers emotion perception as the building block of EI (Joseph and Newman, 2010). Being able to correctly identify emotions based on the cues expressed through the face, voice or body is indeed an important prerequisite to understand and then manage emotions in oneself and others. Emotion recognition ability (ERA) has notably been positively associated with higher interpersonal skills (Hall et al., 2009), empathy and good functioning in work and private relationships (Schlegel et al., 2019).

Most tests designed for assessing ERA rely on affect labeling, i.e., choosing the appropriate emotional label for an emotional expression. In general, unimpaired individuals are very good at this kind of tasks and perform at ceiling when there is no time limit (Wilhelm et al., 2014). In order to investigate individual differences in ERA, different ways to avoid ceiling effects, and therefore being able to rank individuals, can be used: introduce a time limit or make the task more difficult. For instance, in tasks such as the Brief Affect Recognition Test (BART, Ekman and Friesen, 1974) or the Japanese and Caucasian Brief Affective Recognition Test (JACBART, Matsumoto et al., 2000), the presentation time of the stimuli (i.e., prototypical expressions of basic emotions) is limited to 2 s. In the Diagnostic Analysis of Nonverbal Accuracy (DANVA, Nowicki and Carton, 1993), not only the presentation time is limited but also the stimuli vary in intensity and thus in difficulty. Some tests use multimodal and dynamic stimuli (MERT, Bänziger et al., 2009; GERT, Schlegel et al., 2014). They also propose more emotional categories to select from (10 in the MERT and 14 in the GERT), which increases difficulty and allows avoiding ceiling effect. Finally, it is possible to add difficulty to the task by using stimuli that are composites of emotion expressions, such as in the Facial Expression Megamix (Young et al., 1997). In the latter case, the participants have to identify one or both emotional expressions and the presentation time is generally unlimited because the focus is made on accuracy.

In the current study, we present a task aiming at measuring EI_P_ mainly related to the emotion perception facet of EI. Our aims were to shed light on spontaneous processes related to fine-grained recognition of emotion and to allow us to test hypersensitivity related to emotion information. For this purpose, we needed a task that presents complex emotional stimuli (i.e., blended emotional faces) and does not have a ceiling effect. As presented before, in this type of task, emotional stimuli are usually presented for an unlimited time until the participants select a response. In order to make emotion information processing more spontaneous and less thoughtful, we decided to present the emotional stimuli for a limited duration.

We turned to the Test Battery for Measuring the Perception and Recognition of Facial Expressions of Emotion provided in Wilhelm et al. (2014) and selected two tasks that assess emotion categorization (i.e., tasks 4 and 5). These tasks are based on morphed images from two different emotional expressions adjacent on the emotion hexagon and with maximal confusion rates (Calder et al., 1996), such as disgust-anger. Importantly, these morphed images are blends of two emotional expressions displayed on the same face, not composite faces that display one emotion in the upper half of the face and another emotion in the lower half of the face. Contrary to the latter, the former have the advantage of being ecologically valid, reflecting possible emotional expressions that one can encounter in real life, since individuals often feel several emotions at the same time. For instance, surprise and happiness can occur simultaneously when opening a nice gift, or surprise and fear when witnessing a sudden accident on the road. Hence, this type of morphed images was particularly interesting to evaluate hypersensitivity to emotional stimuli.

In tasks 4 and 5 from Wilhelm and colleagues’ battery, the morphed images were presented along the prototypical expressions of the corresponding emotions and the participants had to estimate the ratio of the morphed image on a visual analog scale (task 4) or indicate the prototypical expression to which the morphed image was more similar (task 5). Because we wanted to assess more spontaneous processes related to fine-grained emotion discrimination, in our task, the morphed images were presented by themselves on the screen and for only 1,000 ms. The participants were instructed to determine the correct combination among six possibilities corresponding to the different morphed images categories (i.e., fear-sadness, sadness-disgust, disgust-anger, anger-happiness, happiness-surprise, and surprise-fear). All three facets of participants’ ability-EI (i.e., understanding, management and recognition) were evaluated.

If EI is related to hypersensitivity to emotion information, this should be reflected in higher accuracy in this task for high-compared to low-ability-EI individuals. According to the hypersensitivity hypothesis, individuals high in EI should in principle also be more responsive to emotional signals and this should lead to a stronger capacity to rapidly discriminate complex emotional expressions. Because the task employed involves perception and recognition of emotions, we expected especially the emotion perception facet of EI to be related to it. At the same time, considering that the type of fine-grained discrimination required for this task would provide a fundamental input for in depth emotion understanding and more effective emotion management, we did not exclude positive associations with the other ability-EI facets.

We included a proxy measure for fluid intelligence, to check the extent to which performance in the task was accounted for by individual differences in general reasoning, and a measure of participants’ mood at the time in which they completed the task, to control for potential mood effects on fine-grained emotion discrimination.

## 2. Method

### 2.1. Procedure

The study was conducted in two sessions. The participants took part in a first session where they completed a battery of questionnaires described below. One week later, they were asked to take part in the second session, which consisted in an evaluation of their mood followed by the facial emotion blends discrimination task along with other tasks not reported here.

### 2.2. Participants

Participants (individuals with approval rate of 95% or above) were recruited from the general population on the online platform Prolific. Two-hundred and thirty-nine participants took part in the first session, and 203 participants completed the second session of the study. Because the study was run online and lasted for an hour and a half (both sessions), we followed a strict procedure of exclusion. Participants who did not give correct answers to the attentional checks were removed. We also excluded participants who scored lower than 3 SD from the mean on the Raven and the GERT (less than 4 correct answers in both cases). Hundred and fifty-seven (52 male, 103 female and 2 who indicated “other”) were retained. The participants were aged between 18 and 63 (*M* = 28.9, SD = 9.8). All participants were informed about the course of the study and gave their consent to participate in the study in accordance with procedures and protocols approved by the ethical committee of the University of Geneva. They were remunerated for their participation.

### 2.3. Questionnaires and tests

#### 2.3.1. The shortened Raven’s standard progressive matrices

Participants had to complete 36 items selected from the original Raven SPM (Set B, C, D, Raven et al., 1998). In this task, each item presents a matrix of black and white patterns. Respondents are required to select among 6 or 8 possible choices the correct missing pattern. Responses are scored as correct (1) or incorrect (0). Participants had a 5-min time limit to answer the maximum number of items. The Cronbach alpha was 0.92 in our sample.

#### 2.3.2. The situational test of emotional understanding-brief

The situational test of emotional understanding-brief (STEU-B, Allen et al., 2014) measures respondents’ knowledge of emotions with 19 items that correspond to short scenarios describing situations in which a character experiences an emotion. Respondents are asked to select the appropriate emotion or to answer a question about an aspect of the scenario. For example, for the item “Xavier completes a difficult task on time and under budget. Xavier is most likely to feel?,” the response is “Pride.” Responses are scored as correct (1) and or incorrect (0). The test–retest reliability of the full version of the test is 0.72 (Libbrecht and Lievens, 2012). Cronbach alpha was 0.47 and McDonald’s omega was 0.63 in our sample.

#### 2.3.3. The situational test of emotional management-brief

The situational test of emotional management-brief (STEM-B, Allen et al., 2015) measures the respondents’ knowledge of the strategy to adopt to manage emotions in various situations. In 18 items, respondents are asked to select the most effective way to manage the protagonist’s emotions or the issues they must handle. Responses are scored according to a weight derived from expert ratings. For instance, for the item “Juno is fairly sure his company is going down and his job is under threat. It is a large company and nothing official has been said. What action would be the most effective for Juno?,” the most appropriate response is “Find out what is happening and discuss his concerns with his family.” The test-rest reliability of the full version of the test is 0.85 (Libbrecht and Lievens, 2012). In our sample, Cronbach alpha was 0.62 and McDonald’s omega was 0.65.

#### 2.3.4. The Geneva emotion recognition test short version

The Geneva emotion recognition test short version (GERT-S, Schlegel and Scherer, 2016) measures emotion recognition ability. Respondents see short video clips with sound (duration 1–3 s), in which 10 professional actors express 14 different emotions. After each clip, respondents are asked to choose which of the 14 emotions was expressed by the actor. Responses are scored as correct and incorrect format. Cronbach alpha was 0.78 and McDonald’s omega was 0.81 in our sample.

#### 2.3.5. Brief mood introspection scale

We assessed the participants’ emotional state before the task with the item “Overall, your mood right now is,” from the Brief mood introspection scale (BMIS, Mayer and Gaschke, 1988). Participants answered using a scale ranging from *0* = *Very unpleasant* to *10* = *Very pleasant*.

### 2.4. Facial expressions blends task

The task used in this study (hereafter called FEB task, Facial Expressions Blends) was based on the materials provided in the test battery for measuring the perception and recognition of facial expressions of emotion by Wilhelm et al. (2014). Tasks 4 and 5 from the battery are based on morphed images created from two emotional expressions adjacent on the emotion hexagon, resulting in six emotion continua (happiness-surprise, surprise-fear, fear-sadness, sadness-disgust, disgust-anger, and anger-happiness). Morphs were created for each face separately for five female and five male models. We selected 19 grayscale morphed faces for each emotion continuum, with mixture ratios composed in several steps between 95:5 to 5:95 (more precisely: 95:5, 85:15, 75:25, 70:30, 66:34, 65:35, 62:38, 58:42, 55:45, 54:46, 46:54, 45:55, 42:58, 38:62; 35:65, 34:66, 30:70, 25:75: 15:85, 5:95).

**Figure 1:**
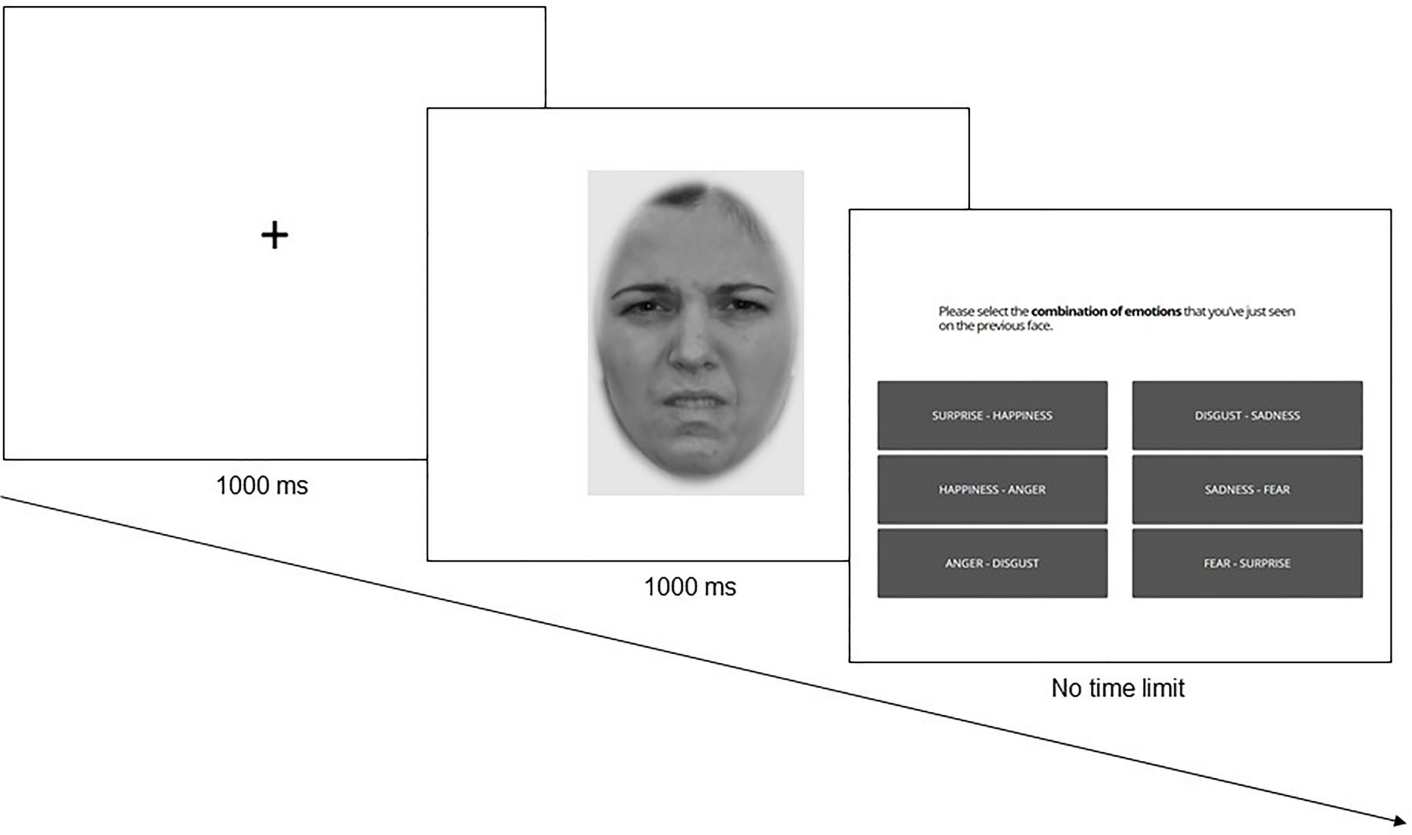
Example of a trial in the FEB task. Morphed face reproduced with permission from Wilhelm et al. (2014).

The FEB task was programmed and run online using Gorilla^1^. For each trial, a fixation cross appeared during 1,000 ms followed by an emotional morphed face presented for 1,000 ms. After the presentation of the emotional morphed face, the six possible emotion combinations were displayed on the screen, and the participants had to indicate which one corresponded to the image previously seen (Figure 1). For instance, if they saw a morphed image of surprise and happiness, they had to select the “SURPRISE – HAPPINESS” combination. The task was composed of 114 trials divided into 3 blocks of 38 trials. Due to the task difficulty, participants had an unlimited time to answer, but they were encouraged to try to answer as fast and as accurately as possible. They were also informed that they would get feedback at the end of the task. Participants had the opportunity to take a break between blocks to ensure that they stayed fully concentrated during the trials. The task started with 6 practice trials.

### 2.5. Data analysis

The relationship between accuracy in the FEB task and EI was analyzed with generalized mixed logistic models in R (R Core Team, 2021). This type of model allows us to analyze binary variables (such as our dependent variable which was correct or incorrect response to each trial) and to account for both within-person (such as in a repeated measures design) and between-person variability. It also allows us to consider all responses, and not only means by condition or by participant. When constructing our models, we followed Baayen et al.’ (2008) procedure and used a forward-approach. In other words, we started with the simplest model, added fixed effects of control variables, and then added fixed effects of explanatory variables (EI facets for example) one a time. We compared the models with a likelihood-ratio test. All continuous independent variables were standardized around the grand mean.

## 3. Results

Hereafter we first present descriptive statistics and correlations between the variables in the study. We then turn to describe how accuracy in the FEB task was influenced by the stimuli characteristics (i.e., emotion combinations and percentages of blends) before analyzing the influence of EI on accuracy.

One participant who scored lower than chance (less than 16.6% of correct responses) in the FEB task was eliminated prior to the analyses. As ERA is generally associated with gender, we also removed both participants that indicated “other” to this question. The analyses were consequently run on 154 participants.

### 3.1. Descriptive statistics

Descriptive statistics and correlations for the variables investigated in the study are shown in Table 1. Accuracy in the task was negatively correlated with age (−0.19) and mood (−0.18) and positively associated with all ability-EI measures (STEU: 0.39, STEM: 0.27, GERT: 0.54) and with fluid intelligence (0.20). Correlations among ability EI measures ranged from 0.29 to 0.56.

**Table 1:** Descriptive statistics and correlations for the variables in the study.

	*M*	SD	1	2	3	4	5	6	7	8	9	10	11	12	13
1. Age	29.1	9.8													
2. Gender (1 = female)	0.7	0.5	0.12												
3. Raven	19.4	5.2	−0.18*	−0.03											
4. STEU	0.61	0.13	−0.07	0.14	0.20*										
5. STEM	0.60	0.12	−0.04	0.07	0.14	0.29***									
6. GERT	0.58	0.15	−0.07	0.11	0.24**	0.56***	0.35***								
7. Mood	6.5	1.9	0.13	0.12	−0.11	−0.16*	−0.08	−0.21**							
8. Accuracy	45.5	8.2	−0.19*	0.14	0.20*	0.39***	0.27***	0.54***	−0.18*						
9. Fe-Sa	37.8	13.4	−0.01	0.12	0.00	0.15	0.25**	0.24**	−0.06	0.28***					
10. Su-Fe	62.2	18.2	−0.13	0.08	0.12	0.32***	0.16*	0.40***	−0.06	0.74***	0.21**				
11. Sa-Di	38.6	14.5	−0.13	0.13	0.19*	0.30***	0.14	0.31***	−0.13	0.61***	−0.01	0.32***			
12. An-Ha	22.8	12.6	−0.22**	−0.05	0.07	0.07	0.07	0.07	−0.19*	0.36***	−0.09	0.11	0.15		
13. Di-An	49.7	17.3	−0.17*	0.13	0.21**	0.29***	0.21*	0.41***	−0.13	0.63***	0.04	0.38***	0.27***	0.14	
14. Ha-Su	62.2	15.5	0.00	0.03	0.04	0.13	0.06	0.23**	−0.02	0.46***	−0.10	0.20*	0.21**	0.02	0.09

Spearman correlations were calculated because most variables were not normally distributed. For gender, the point-biserial correlations are reported. STEU, situational test of emotion understanding; STEM, situational test of emotion management; GERT, Geneva emotion recognition test; Accuracy, percentage of correct responses in the FEB task; Sa-Fe, percentage of correct responses for the sadness-fear blends; Fe-Su, percentage of correct responses for the fear-surprise blends; Sa-Di, percentage of correct responses for the sadness-disgust blends; An-Ha, percentage of correct responses for the anger-happiness blends; Di-An, percentage of correct responses for the disgust-anger blends; Ha-Su, percentage of correct responses for the happiness-surprise blends. *p < 0.05, **p < 0.01, ***p < 0.001.

**Figure 2:**
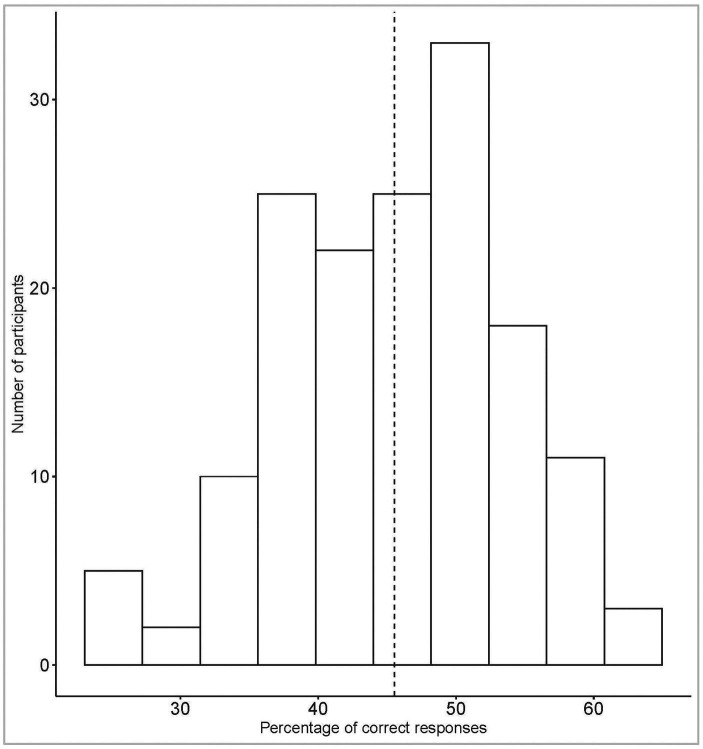
Distribution of percentage of correct responses in our sample (*N* = 154).

Figure 2 shows the participants’ accuracy. The distribution reveals that the task was quite difficult: the percentage of correct responses varied between 25 and 63. The distribution was normal (*W* = 0.99, *p* = 0.28) and reliability estimates were good (*α* = 0.74, *ω* = 0.81).

**Figure 3:**
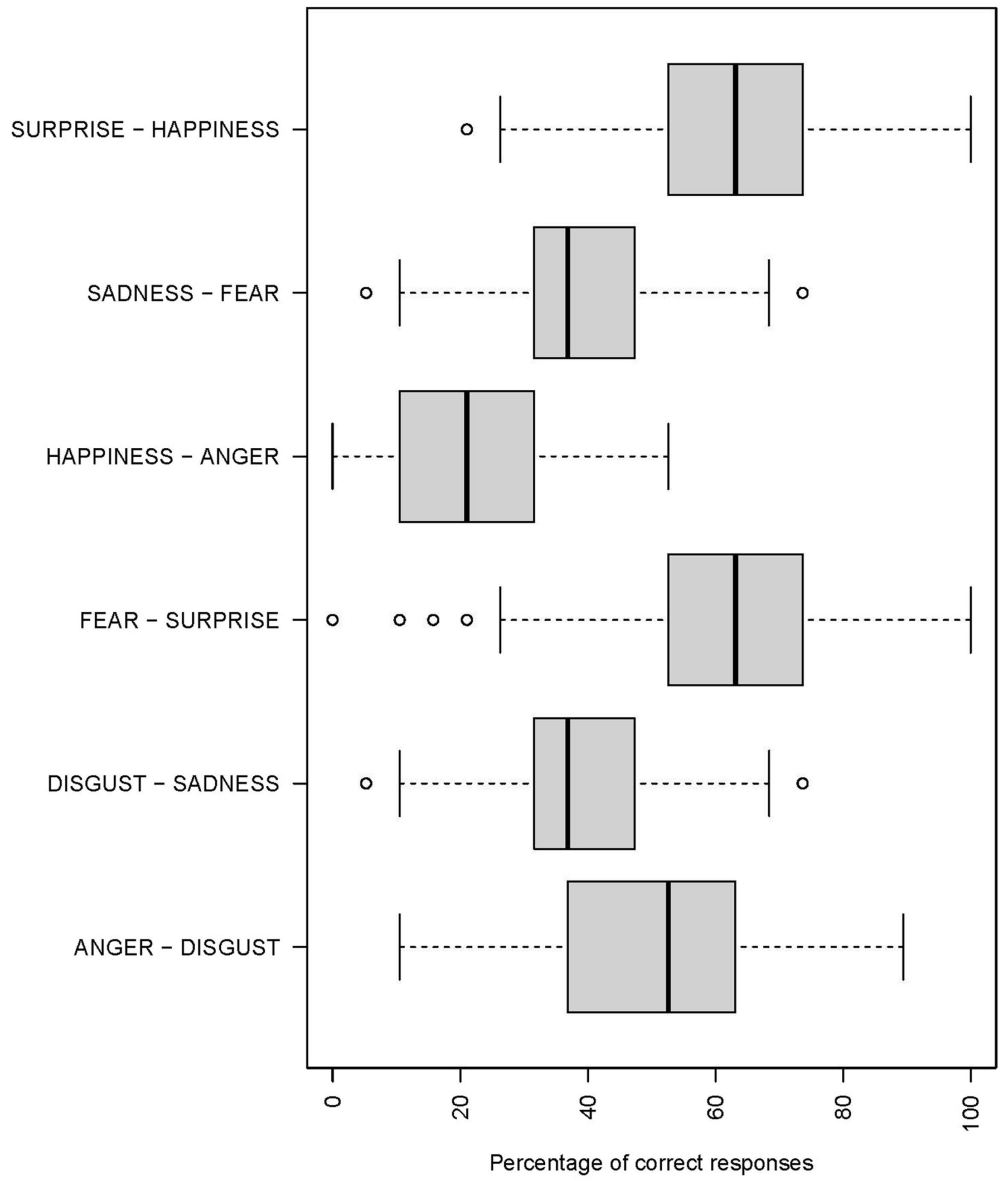
Percentage of correct responses as a function of emotions combinations.

When looking at accuracy in function of emotion combination (Figure 3), combinations of surprise-fear (*M* = 62.2, SD = 18.2) and happiness-surprise (*M* = 62.2, SD = 15.5) were better recognized than disgust-anger (*M* = 49.7, SD = 17.3), which was better recognized than sadness-disgust (*M* = 38.6, SD = 14.5) and fear-sadness (*M* = 37.8, SD = 13.4). Anger-happiness (*M* = 22.8, SD = 12.6) was the least recognized combination [*F*(5,765) = 180.93, *p* < 0.001]. Generally, accuracy for the different emotion blends was correlated with EI facets, except for the anger-happiness combination with was not associated with any facet.

**Figure 4:**
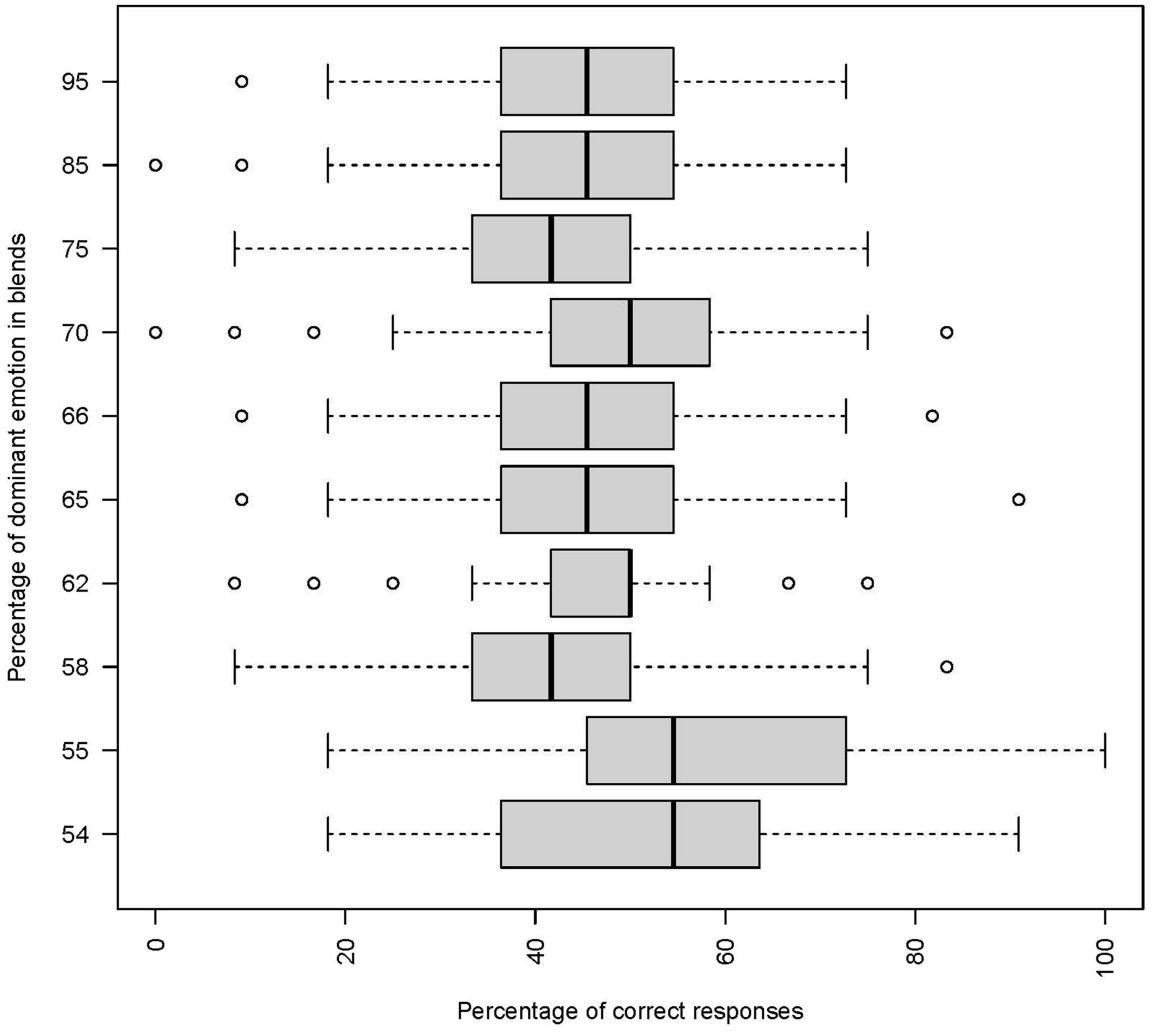
Percentage of correct responses as a function of percentage of the dominant emotion in emotion blends.

Regarding the percentage of blends (Figure 4), accuracy was the highest when the prevailing emotion corresponded to 54–55% and decreased with increasing contribution of the main emotion [*F*(9,1,377) = 27.24, *p* < 0.001]. Hence, expressions in which an emotion was stronger and the other very subtle were the most difficult to evaluate.

### 3.2. Relationship with EI

As described above, our data were analyzed with generalized mixed logistic models. In the first model, we included fixed effects of age, gender, fluid intelligence and mood at testing time, and a random intercept by participant. This model returned significant effects of age (OR = 0.93, 95%CI [0.88-0.98], *p* = 0.005), mood (OR = 0.94, 95%CI [0.90-0.99], *p* = 0.02), and gender (OR = 1.15, 95%CI [1.03-1.27], *p* = 0.01) but no effect of fluid intelligence. In order to verify that the performance was not influenced by motivation or fatigue effects, we added block in the model, which did not improve it (*χ^2^* = 1.06, df = 2, *p* = 0.59).

When adding the STEU score to the first model, the model improved significantly (*χ^2^* = 24.11, df = 1, *p* < 0.001) and showed that individuals high on emotion understanding were more likely to give correct responses in the task (OR = 1.13, 95%CI [1.08–1.19], *p* < 0.001). We then added the STEM score and the model improved again (*χ^2^* = 5.62, df = 1, *p* = 0.018), showing that emotion management also predicted accuracy (OR = 1.06, 95%CI [1.01–1.11], *p* = 0.017). We finally added the GERT score and the model improved further (*χ^2^* = 25.03, df = 1, *p* < 0.001). In this last model, only age and emotion recognition were significant predictors of accuracy in the task. Increasing age was associated with a decrease in the likelihood to choose a correct answer (OR = 0.94, 95%CI [0.90–0.98], *p* = 0.003) whereas increasing emotion recognition ability increased this likelihood (OR = 1.16, 95%CI [1.09–1.22], *p* < 0.001). Neither gender nor fluid intelligence played a role in the models when the different facets of EI were added (see Table 2 for outputs of models).

**Table 2:** Mixed logistics models testing the influence of EI on accuracy in the FEB task.

	Model 1			Model 2			Model 3			Model 4		
Predictors	Odds ratios	CI	*p*	Odds ratios	CI	*p*	Odds ratios	CI	*p*	Odds ratios	CI	*p*
Intercept	0.76	0.70–0.83	<0.001	0.78	0.72–0.85	<0.001	0.78	0.73–0.85	<0.001	0.79	0.74–0.85	<0.001
Age	0.93	0.88–0.98	0.005	0.93	0.89–0.97	0.002	0.93	0.89–0.97	0.002	0.94	0.90–0.98	0.003
Raven	1.05	0.99–1.10	0.080	1.02	0.98–1.07	0.338	1.02	0.97–1.07	0.471	1.01	0.96–1.05	0.805
Mood	0.94	0.90–0.99	0.024	0.96	0.92–1.01	0.099	0.96	0.92–1.01	0.088	0.98	0.94–1.02	0.371
Gender, female	1.15	1.03–1.27	0.011	1.10	0.99–1.21	0.066	1.09	0.99–1.21	0.072	1.08	0.98–1.18	0.105
STEU				1.13	1.08–1.19	<0.001	1.11	1.06–1.17	<0.001	1.04	0.99–1.09	0.151
STEM							1.06	1.01–1.11	0.017	1.03	0.98–1.07	0.277
GERT										1.16	1.09–1.22	<0.001
Random effects												
*σ* ^2^	3.29			3.29			3.29			3.29		
*τ*_00_	0.06 _PARTICIPANT_			0.05 _PARTICIPANT_			0.04 _PARTICIPANT_			0.03 _PARTICIPANT_		
ICC	0.02			0.01			0.01			0.01		
*N*	154			154			154			154		
Observations	17,556			17,556			17,556			17,556		
Marginal *R*^2^/Conditional *R*^2^	0.005/0.023			0.009/0.023			0.010/0.023			0.013/0.023		

Odds ratios correspond to the exponential function of the regression coefficient from the logistic regression. STEU, situational test of emotion understanding; STEM, situational test of emotion management; GERT, Geneva emotion recognition test.

We then investigated whether ability-EI interacted with the percentage of emotion blends when predicting the participants’ accuracy. In other words, we tested whether the influence of the different EI facets depended on the percentage of emotion blends of the stimuli.

**Figure 5:**
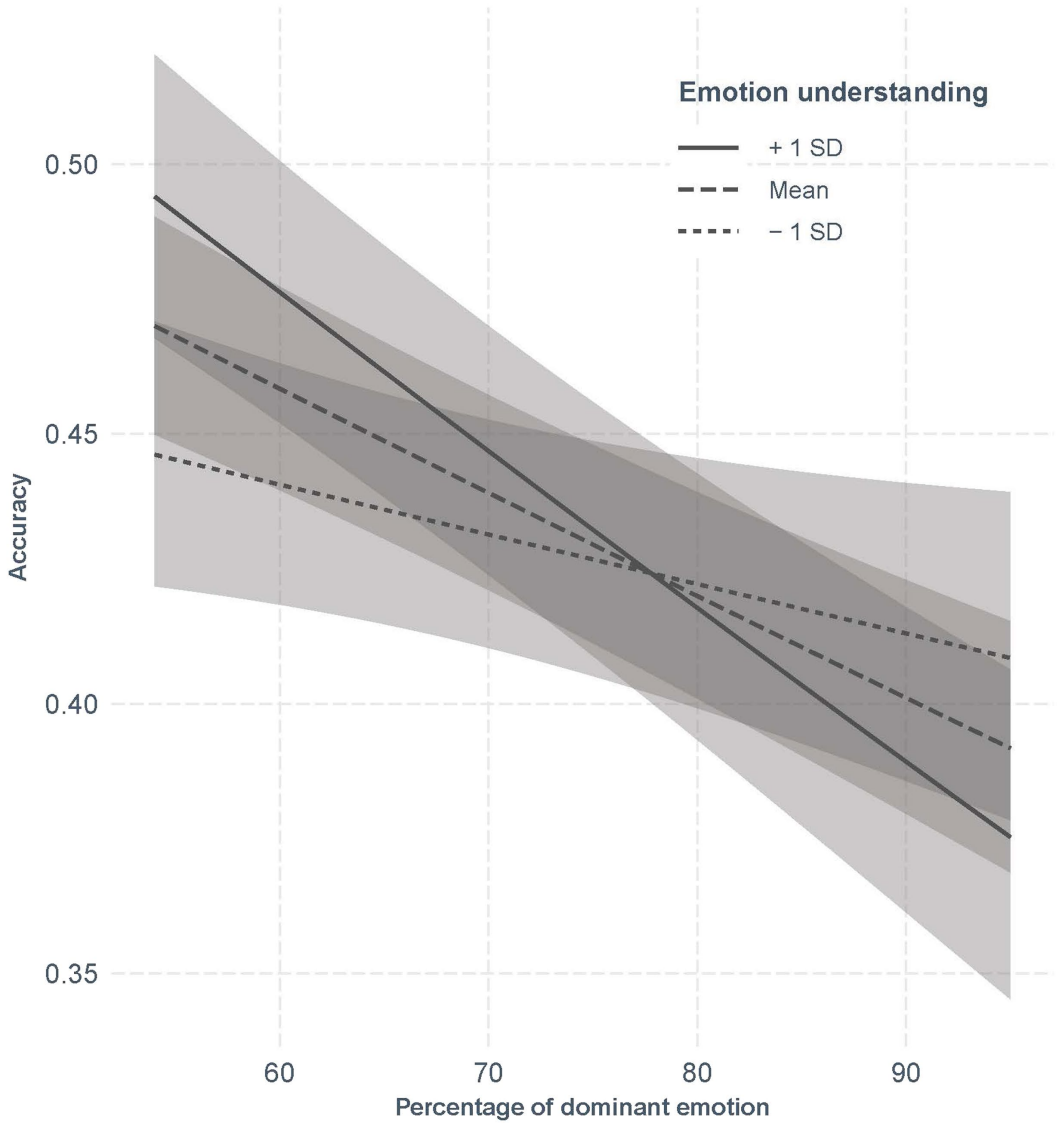
Accuracy in the task in function of emotion understanding and percentage of dominant emotion in emotion blends.

In order to do so, we added the main effect of percentage of emotion blends to model 4, which improved the model (*χ^2^* = 40.72, df = 1, *p* < 0.001). In the next model, we included the interaction between STEU and percentage of emotion blends and the model improved further (*χ^2^* = 10.70, df = 1, *p* = 0.001) (Table 3). In this model, in addition to the effects of age and emotion recognition, there was also an effect of percentage of emotion blends (OR = 0.91, 95%CI [0.88–0.94], *p* < 0.001) which showed that with increasing percentage of the main emotion, participants were less likely to choose the correct emotion combination. There was also an interaction between STEU and percentage of emotion blends (OR = 0.95, 95%CI [0.92–0.98], *p* = 0.001). Simple slopes analysis with the Johnson-Neyman procedure revealed that the effect of STEU was only significant for combinations with the dominant emotion below 65%. In other words, for less difficult items, individuals high on emotion understanding had higher accuracy than those low on this facet. For more difficult items however, there was no difference between individuals depending on their level of emotion understanding (Figure 5).

**Table 3:** Mixed logistics models testing the influence of EI and mixture ratio on accuracy in the FEB task.

	Model 5			Model 6			Model 7		
Predictors	Odds ratios	CI	*p*	Odds ratios	CI	*p*	Odds ratios	CI	*p*
(Intercept)	0.79	0.74–0.85	<0.001	0.79	0.74–0.85	<0.001	0.79	0.74–0.85	<0.001
Age	0.94	0.90–0.98	0.003	0.94	0.90–0.98	0.003	0.94	0.90–0.98	0.003
Gender, female	1.08	0.98–1.18	0.105	1.08	0.98–1.18	0.105	1.08	0.98–1.18	0.105
STEU	1.04	0.99–1.09	0.155	1.04	0.99–1.09	0.156	1.04	0.99–1.09	0.155
STEM	1.03	0.98–1.07	0.277	1.03	0.98–1.07	0.273	1.03	0.98–1.07	0.277
GERT	1.16	1.10–1.22	<0.001	1.16	1.10–1.22	<0.001	1.16	1.09–1.22	<0.001
Mood	0.98	0.94–1.02	0.373	0.98	0.94–1.02	0.372	0.98	0.94–1.02	0.373
Raven	1.01	0.96–1.05	0.805	1.01	0.96–1.05	0.805	1.01	0.96–1.05	0.805
Ratio	0.91	0.88–0.94	<0.001	0.91	0.88–0.93	<0.001	0.91	0.88–0.94	<0.001
STEU × Ratio	0.95	0.92–0.98	0.001	0.94	0.91–0.97	<0.001	0.95	0.92–0.99	0.013
STEM × Ratio				1.03	0.99–1.06	0.111			
GERT × Ratio							0.99	0.96–1.03	0.778
Random effects									
*σ* ^2^	3.29			3.29			3.29		
*τ*_00_	0.03 _PARTICIPANT_			0.03 _PARTICIPANT_			0.03 _PARTICIPANT_		
ICC	0.01			0.01			0.01		
*N*	154			154			154		
Observations	17,556			17,556			17,556		
Marginal *R*^2^/Conditional *R*^2^	0.017/0.026			0.017/0.027			0.017/0.026		

Odds ratios correspond to the exponential function of the regression coefficient from the logistic regression. STEU, situational test of emotion understanding; STEM, situational test of emotion management; GERT, Geneva emotion recognition test.

Adding the interaction between STEM and ratio or between GERT and ratio did not improve the model (Table 3). Finally, we also tested whether emotion combination interacted with the different EI facets when predicting accuracy, which was not the case.

## 4. Discussion

In this paper, we aimed to create an EI_P_ task that would measure fine-grained discrimination of emotional expressions. We also wanted to assess whether individuals who are high on EI show stronger reactivity to emotion information and hence better performance than low EI individuals, in line with the hypersensitivity hypothesis (Fiori and Ortony, 2021). For this purpose, we created a task based on blends of emotional expressions in which participants were presented with the pictures for 1,000 ms before having to choose the correct combination of emotions among 6 possibilities. These specific choices aimed at making the task more spontaneous than usual emotion discrimination tasks while maintaining a high level of difficulty. Hypersensitivity in fine-grained discrimination of emotions was operationalized as high accuracy in the task.

Accuracy in the task was influenced by the characteristics of the stimuli. First, some emotion combinations were generally more recognizable than others, suggesting that certain emotion combinations are easier to categorize. Interestingly, emotion combinations displaying surprise seemed easier to recognize, as shown by the higher number of correct responses associated with them, probably because of the specificity of the surprise expression that was combined to emotions that have quite opposite specificities (i.e., happiness and fear).

Second, the percentage of emotion blends influenced accuracy. Blends of emotional expressions were more difficult to categorize as the percentage of the dominant emotion increased. This is not surprising, as the participants had in total six possibilities to choose from, with two possibilities pertaining to each emotion. With balanced percentages of emotions, it might be easier to find cues pertaining to both emotions displayed and then select the correct combination. A higher percentage of the dominant emotion implies less cues for detecting the second emotion, which might lead to a choice based on chance between the emotion combinations containing the dominant emotion.

Interestingly, in task 4 of Wilhelm et al. (2014), in which participants had to evaluate the percentage of emotion blends, they found that accuracy increased with increasing percentage of the dominant emotion, which is the opposite of what was found in this study. This can be explained by differences in the tasks. Perhaps we could have found a similar effect if we had asked our participants to identify both emotions independently (i.e., select the first emotion among six possibilities and then the second emotion among the same possibilities). Of note, our task was initially designed in this way, but it was too difficult, and did not seem to fully capture individual differences in fine-grained emotion discrimination; for this reason, we chose to present the possible emotion combinations as responses in the task. The distribution of the scores did not show any floor or ceiling effects and allowed us to observe individual differences in EI_P_ related to emotion discrimination.

Regarding the participants’ characteristics, age was associate with a decrease in accuracy, which is in line with previous findings showing a decline in ERA with age (e.g., Ruffman et al., 2008; Schlegel et al., 2019). Supporting previous findings that females have a small advantage in ERA (Schlegel et al., 2014; Thompson and Voyer, 2014; Schlegel, 2019), sex was associated with accuracy in the task, but only when other variables (i.e., age, EI level) were not controlled for.

Turning to the main interest of this study, we found that the FEB task was associated with all facets of EI, which suggests that this task indeed measures a component of EI. The fact that emotion understanding, emotion management and emotion recognition predicted accuracy in the FEB task beyond control variables further suggests that individuals high on EI also have higher emotion information processing skills (i.e., EI_P_) related to fine-grained discrimination of emotional expressions. Of note, when including emotion recognition in the model, the influence of emotion understanding and emotion management disappeared, and only emotion recognition predicted the performance. This supports previous findings showing that, in a go/nogo task, participants with higher emotion perception ability were better at discriminating emotions (Gutiérrez-Cobo et al., 2017). This could also be explained by the fact that the three facets of EI are correlated. For this reason, emotion understanding and emotion management did not predict the performance beyond the influence of emotion recognition. Still, the fact that they individually were associated with accuracy in the discrimination task supports the idea that hypersensitivity related to perceptual processing of emotional expression is associated with all facets of EI. In addition, when controlling for emotion recognition level, we found that emotion understanding still predicted accuracy for less difficult stimuli.

All in all, this study supports the idea that individuals high in EI have higher emotion processing skills at the emotion perception stage of information processing. It also shows that a form of sensibility (or emotional hypersensitivity) in fine-grained discrimination of emotional expressions is associated with all facets of EI. This is consistent with the hypersensitivity hypothesis and the idea that perceptual processes, such as those captured by the EI emotion perception facet, underlie all EI abilities (Mayer et al., 2008).

Hence, we believe the task employed in the current study might be employed as a new measure of the EI_P_ component. The FEB task involves complex stimuli presented for a limited amount of time, which we think evaluates fast information processing and can describe more effectively spontaneous processes involved in emotion perception. As such, accuracy in the FEB task might predict different outcomes (i.e., less thoughtful behaviors) as those related to other emotion recognition tasks such as the GERT (Schlegel et al., 2014) for instance. Yet, further research is needed to fully test the validity of the task, especially its incremental validity.

Despite the encouraging results obtained in the current study, we think that the FEB task presented here could be improved in several ways. First, the task was rather difficult as demonstrated by the participant’s performance, which might have dampened their motivation throughout the experiment. However, as there was no block effect, we are confident that this was not the case. Second, the difficulty of the task could have diminished the role of emotion understanding and emotion management facets on performance. There was indeed an effect of emotion understanding for less difficult items that was not observable for more difficult ones. It is possible that with a less difficult task (with longer presentation time for instance), or with more items in the task (which would increase power) the relationship between emotion understanding and accuracy would be stronger beyond the effect of emotion recognition.

Another limitation concerns the fact that we did not control for crystallized intelligence in our study. Recent findings (Davis et al., 2021) have indeed shown that the emotion understanding and emotion management facets of EI did not predict performance beyond this type of intelligence in an emotion recognition task. Even though we reckon that crystallized intelligence might play a lesser role than fluid intelligence in the FEB task (notably because of the fast presentation of the stimuli), it would be important to include it in further research.

Finally, further research is also still needed to determine whether EI_P_ related to emotion perception as measured in this study explains additional variability in emotionally intelligent behavior. In a similar way as proposed by Fiori et al. (2022), it would be necessary to investigate whether performance in such a task adds to classic ability-EI measures when explaining real life outcomes. We can think for instance of a study measuring personality traits, trait-EI and fluid and crystallized intelligence as controls, in addition to classical ability-EI (EI_K_) and the FEB task (EI_P_) presented here and assess their respective influence on an outcome such as performance in a negotiation task (i.e., where fine-grained discrimination of emotions is crucial). Another interesting line of research would be to investigate whether better emotion information processing skills are related to more emotional activation during the task. It would indeed be possible that those individuals who perform better at emotion processing tasks also respond more strongly to emotion.

In sum, in this study we aimed at testing a type of emotion information processing task related to fine-grained discrimination of emotional expressions that could be employed as a measure of the EI_P_ component of emotional intelligence. This task is different from previous ability EI tasks because it taps into more spontaneous processing of emotion information, it includes complex emotional expressions made of morphed blends of emotions, and it is quite difficult, although in a way that allows measuring individual differences in EI_P_. Hence, the FEB task presented here could be a valuable alternative to existing EI tests for those researchers interested to capture more spontaneous emotional behavior, such as when people react to stressful situations without having much time or cognitive resources to think about what to do, or when interacting with other individuals with little time for processing others’ emotional reactions. Although results were generally encouraging, further research is needed to ascertain the validity of such task as a measure of individual differences in EI and as a new test that may predict additional variance on top of existing ability EI tests.

## Data availability statement

The raw data supporting the conclusions of this article are available on OSF, https://osf.io/7gdxz/.

## Ethics statement

The studies involving human participants were reviewed and approved by the ethical committee of the University of Geneva. The patients/participants provided their written informed consent to participate in this study.

## Author contributions

CG: conceptualization, methodology, formal analysis, writing: original draft, review and editing. MN-d-F: conceptualization, methodology, formal analysis, writing: review and editing. OW: resources, writing: review and editing. MF: funding acquisition, conceptualization, writing: review and editing. All authors contributed to the article and approved the submitted version.

## Funding

The research presented in this manuscript was supported by a grant from the Swiss National Science Foundation (10001C_192443) awarded to MF.

## Conflict of interest

The authors declare that the research was conducted in the absence of any commercial or financial relationships that could be construed as a potential conflict of interest.

## Publisher’s note

All claims expressed in this article are solely those of the authors and do not necessarily represent those of their affiliated organizations, or those of the publisher, the editors and the reviewers. Any product that may be evaluated in this article, or claim that may be made by its manufacturer, is not guaranteed or endorsed by the publisher.

## Footnotes

^1^ https://gorilla.sc
